# The Potential Circular RNAs Biomarker Panel and Regulatory Networks of Parkinson’s Disease

**DOI:** 10.3389/fnins.2022.893713

**Published:** 2022-05-13

**Authors:** Yousheng Xiao, Hongchang Chen, Jiajia Liao, Qinxin Zhang, Honghu He, Jiang Lei, Jinjun Huang, Qiang Ouyang, Yuefei Shen, Jin Wang

**Affiliations:** ^1^Department of Neurology, The First Affiliated Hospital of Guangxi Medical University, Nanning, China; ^2^Department of Neurology, Minzu Hospital of Guangxi Zhuang Autonomous Region, Nanning, China; ^3^Department of Rehabilitation, Guiping People’s Hospital, Guiping, China

**Keywords:** circular RNA, Parkinson’s disease, biomarker, regulatory networks, diagnosis, bioinformatics

## Abstract

Parkinson’s disease (PD) is a progressive neurodegenerative disease. It has been reported that circular RNAs (circRNAs) play important roles in several neurological diseases. However, the role and regulatory networks of circRNAs in PD are still largely unclear. In this study, we first compared the global expression level of circRNAs from patients with PD and controls using microarray, then the candidate circRNAs were validated in another PD cohort. The possible functions of these candidate circRNAs were analyzed using Gene Ontology (GO) analyses and Kyoto Encyclopedia of Genes and Genomes (KEGG) analyses, and the regulatory networks of these candidate circRNAs were constructed through circRNA–miRNA–mRNA regulatory networks, protein–protein interaction (PPI) networks, and transcription factor-circRNA networks. The results indicated that hsa_circRNA_101275, hsa_circRNA_103730, and hsa_circRNA_038416 were significantly more highly expressed in patients with PD, while hsa_circRNA_102850 was lower expressed in patients with PD when compared with controls. A circRNA panel combining the four differentially expressed circRNA showed a high diagnostic ability to distinguish patients with PD from controls (AUC = 0.938). Furthermore, GO and KEGG analysis showed these candidate circRNAs were enriched in PI3K–Akt and MAPK signaling pathways. We established circRNA–miRNA–mRNA regulatory networks and identified 10 hub genes (*ESR1*, *PTEN*, *SHC1*, *IGF1R*, *SMAD2*, *KRAS*, *MDM2*, *HIF1A*, *BMP4*, and *ACVR2B*) were closely related to PD by using PPI network analysis. Besides, these circRNAs were predicted to be regulated through tyrosine hydroxylase (TH)-relevant transcription factors such as *GATA2* and *GATA3*. In conclusion, our results suggest that the circRNA panel and the established circRNA–miRNA–mRNA regulation networks might provide potential novel biomarkers and therapeutic targets for PD.

## Introduction

Parkinson’s disease (PD) is one of the most common neurodegenerative diseases. It is generally considered that the prevalence of PD ranges from 100 to 200 per 100,000 people and affects 1% of the population over the age of 60 years (Tysnes and Storstein, 2017). The loss of dopaminergic neurons in the substantia nigra compact is considered the neuropathological characteristic of PD. PD is characterized by motor symptoms including tremors, rigidity, postural instability, bradykinesia, and non-motor characteristics such as autonomic nervous dysfunction, sleep disorders, and cognitive and mental disorders (Chen et al., 2020).

As we know, PD is diagnosed mainly based on clinical features. Therefore, it is extremely difficult to make a precise diagnosis in its early stage. Although studies have focused on the identification of potential biomarkers for PD, such as neuroimaging, cerebrospinal fluid, serum, and saliva biomarkers, there is still a lack of reliable biomarkers for the early diagnosis of PD (Ba and Martin, 2015; Marques et al., 2017; Thobois et al., 2019). Therefore, it is of great interest to identify newly non-invasive biomarkers and therapeutic targets for PD.

Circular RNAs (circRNAs) are recently discovered non-coding RNAs (ncRNAs) that are generated through backsplicing, in which the 5′ and 3′ ends of the RNA molecule are covalently connected (Kristensen et al., 2019). They have high stability and resistance to degradation due to their circular shape and absence of a free end (Suzuki et al., 2006). It has been demonstrated that circRNAs exhibit distinct properties such as tissue specificity and stability in both intracellular and extracellular contexts, which are readily accessible and quantifiable in bodily fluids such as cerebrospinal fluid, blood, plasma, and saliva (Wang et al., 2021). These characteristics of circRNAs suggest that they may be used as novel non-invasive biomarkers. In fact, several studies have identified a few circRNAs in neurodegenerative diseases such as Alzheimer’s disease (Akhter, 2018) and multiple system atrophy (Mehta et al., 2020). It is worth mentioning that there are currently few reports on circRNAs in PD. Although some circRNAs have been identified that may be dysregulated in PD (Zhong et al., 2021), the networks and underlying mechanisms involved in circRNA-mediated regulation of PD still need to be explored.

In this study, first, the global expression levels of the circRNAs in patients with PD and healthy controls were compared through microarray, then the results were validated in another independent PD cohort. Thereafter, candidate circRNAs were further analyzed. The potential miRNAs sponged by candidate circRNAs and their target genes were analyzed by bioinformatic analysis. Furthermore, the circRNA–miRNA–mRNA regulatory networks, protein–protein interaction, and transcription factor–circRNA interaction were constructed for predicting the biological function of the circRNAs for PD.

## Materials and Methods

### Patients and Samples

We recruited 63 patients With PD and 60 healthy individuals From the First Affiliated Hospital of Guangxi Medical University Between January 2019 and June 2021. Patients With PD Were diagnosed independently by Two neurologists based on the United Kingdom Parkinson’s Disease Brain Bank Criteria (Hughes et al., 1992). the control groups consisted of healthy people matched With PD in terms of gender and age and Had no known neurological disease, comorbidities, or PD family history. the Ethics Committee of the First Affiliated Hospital of Guangxi Medical University approved this study. All participants provided informed consent. the clinical and demographic characteristics of all participants Are outlined in Table 1. A total of 123 peripheral blood samples Were obtained From patients With PD and healthy individuals and stored vertically at −80°C until total RNA isolation.

**TABLE 1:** The top 5 upregulated and downregulated differently expressed circRNAs in PD group and control group.

CircRNAs	Regulation	FC (abs)	Chromosome	circRNA_type	Alias	Source
hsa_circRNA_406587	Up	2.5394136	chr5	Intronic		25070500
hsa_circRNA_038416	Up	2.4182189	chr16	Exonic	hsa_circ_0038416	circBase
hsa_circRNA_103730	Up	2.2940354	chr4	Exonic	hsa_circ_0005654	circBase
hsa_circRNA_103224	Up	2.2144709	chr22	Exonic	hsa_circ_0063329	circBase
hsa_circRNA_102850	Up	2.1595253	chr2	Exonic	hsa_circ_0001081	circBase
hsa_circRNA_402563	Down	2.9645622	chr20	Exonic		25242744
hsa_circRNA_082317	Down	2.473676	chr7	Exonic	hsa_circ_0082317	circBase
hsa_circRNA_104327	Down	2.130632	chr7	Exonic	hsa_circ_0001971	circBase
hsa_circRNA_406019	Down	2.0713993	chr2	Sense overlapping		25070500
hsa_circRNA_101275	Down	2.0231739	chr13	Exonic	hsa_circ_0030428	circBase

*Hsa, homo sapiens; circRNA, circular RNA; FC, fold-change; abs, absolute ratio; chr, chromosome; up, upregulation; down, downregulation.*

RNA Isolation and Real-Time Quantitative Polymerase Chain ReactionTRIzol reagent kit (Invitrogen, Takara, Japan) Was used to extract total RNA From blood samples according to the protocol provided by the manufacturer. After extraction, total RNA Was reverse transcribed Into cDNA (RR047A, Takara, Japan). the RT-qPCR Was performed on the ABI PRISM 7500 (RR820A, Takara, Japan). Glyceraldehyde 3-phosphate dehydrogenase (GAPDH) Was used as an internal reference to normalize the qPCR data. the 2^–ΔΔCT^ method Was used to calculate the relative expression of circRNAs. the primers Are described in Supplementary Table 1.

### CircRNA Microarray and Analysis

Sample preparation and microarray hybridization were performed according to the protocol of the Arraystar. KangChen Bio-tech Inc. (Shanghai, China) carried out the Arraystar Human circRNA Array version 2 analysis. To summarize, circRNAs from each sample were enriched by removing linear RNAs and then amplified following the manufacturer’s instructions. The labeled circRNAs were hybridized onto the Arraystar and scanned by the Agilent Scanner. CircRNAs exhibiting a fold change (FC) ≥1.5 with a *p*-value ≤ 0.05 were considered significantly different. Volcano plot filtering and fold change filtering were used to identify differentially expressed circRNAs. The distinct circRNA expression patterns among samples were analyzed using hierarchical clustering. The circRNA microarray raw data have been stored in the Gene Expression Omnibus (GEO) (series number: GSE198273).

### Criteria for Selecting circRNA for Further Bioinformatics Analysis

The area under the curve (AUC) of the receiver operating characteristics (ROC) is used to assess the accuracy of biomarkers. The AUC is near 1, which means an ideal model (Pepe et al., 2009). The following guidelines are often used to estimate the specificity and sensitivity of a biomarker, e.g., excellent scored 0.9–1.0; good scored 0.8–0.9; acceptable scored 0.7–0.8; while poor scored 0.6–0.7 and fail scored 0.5–0.6 (Xia et al., 2013; Ren et al., 2018). The circRNA level with an AUC > 0.70 was selected to proceed forward with bioinformatics analysis to investigate potential functions.

### CircRNA–miRNA–mRNA Interaction Networks

We constructed circRNA–miRNA–mRNA networks in Cytoscape based on circRNA microarray data and the RT-qPCR results. The circRNA–miRNA interaction was predicted using Arraystar’s miRNA target prediction software. The Cytoscape plug-in ClueGO (2.5.8) and CluePedia (1.5.8) were used to predict target genes of the top 5 putative miRNAs for the candidate circRNAs, and the top 200 target genes were screened for the construction of the circRNA–miRNA–mRNA networks.

### Functional Enrichment Analyses

Gene Ontology (GO) enrichment analysis was used to establish functional annotations of miRNA target genes. Kyoto Encyclopedia of Genes and Genomes (KEGG) enrichment analysis was used to explore the functions of these differentially expressed genes. Statistical analyses and visualizations were conducted in R 3.6.3 by using the clusterProfiler package and the org.Hs.eg.db package.

### Protein–Protein Interaction Network Analysis

The protein–protein interaction (PPI) network was created by using the Retrieval of Interacting Genes (STRING) online tool to evaluate the interactions among the target genes that were identified in the circRNA-miRNA-mRNA networks. The hub genes were screened from the PPI network using the cytoHubba plugin.

### Transcription Factor Prediction

To examine the upstream regulation mechanism of the candidate circRNAs, transcription factors for circRNAs were screened using the TRCirc database^1^.

### Statistical Analyses

Statistical analyses were conducted using SPSS 20.0 software (IBM, Armonk, NY, United States). The continuous variables were presented as mean ± standard deviation (SD). The differences in age and sex between patients with PD and healthy controls were assessed using the chi-square (χ^2^) test or Student’s *t*-test. ROC curves were used to assess the potential biomarkers of the candidate circRNAs. Two-tailed *p*-values < 0.05 were considered significantly difference.

## Results

### A Summary of the circRNA Microarray Data Profile

**FIGURE 1:**
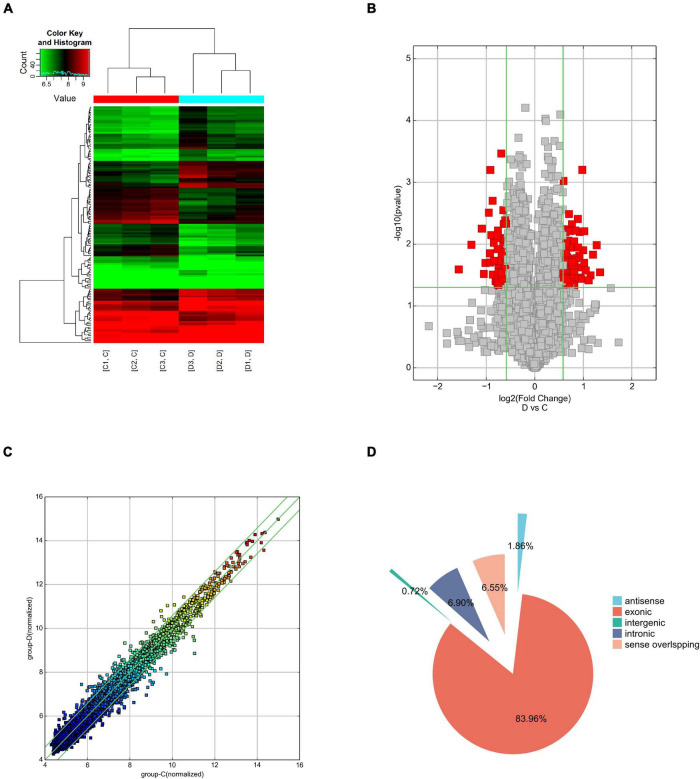
Overview of the circular RNA (circRNA) microarray expression data. **(A)** Hierarchical clustering of the differentially expressed circRNA expression data in the PD group and control group, and upregulated and downregulated circRNAs are colored in red and green, respectively. **(B)** Volcano plot of the differentially expressed circRNAs in the PD group and control group. The vertical green lines indicate a 2.0-fold (log2-scaled) up and down, respectively. The horizontal green line represents *P* = 0.05 (–log10-scaled). The red squares in the plot represent the differentially expressed circRNAs with statistical significance. **(C)** The scatter plot reveals the variation in circRNA expression between the PD group and control group. The *X* and *Y* values on the scatter plot are the average normalized signal values, shown on a log2 scale. The green fold-change lines represent 2-fold changes, thus circRNAs above or below the green lines indicate >2.0-fold upregulation or downregulation. **(D)** Classification of circRNAs in the PD group and control group.

Human circRNA microarray was used to screen dysregulated circRNAs from three patients with PD and three healthy controls. The mean age was 62.67 ± 8.36 years for the PD group and 63.00 ± 8.67 years for controls. Both groups have two male participants and one female participant. The expression features of the dysregulated circRNAs were depicted in a two-dimensional hierarchical clustering heatmap (Figure 1A). A volcano plot (Figure 1B) and a scatter diagram (Figure 1C) were also utilized to show the differences in circRNA expression between both groups. According to microarray data analyses, a total of 10,145 circRNAs were identified, with 189 antisense (1.86%), 8,518 exonic (83.96%), 73 intergenic (0.72%), 700 intronic (6.90%), and 665 sense overlapping (6.55%) (Figure 1D). After scanning and normalization, 139 differentially expressed circRNAs were identified with a threshold of fold-change (FC) absolute value ≥1.5 and *P* < 0.05. Of them, 78 circRNAs were upregulated and 61 were significantly downregulated. The top 5 upregulated and downregulated circRNAs are presented in Table 2.

**TABLE 2:** Demographic and clinical characteristics of PD group and healthy controls.

Variables	PD (*n* = 63)	Healthy control (*n* = 60)	Comparison
Gender (male/female)	35/28	33/27	*x*^2^ = 0.004; *p* = 0.951
Age (years)^a^	60.16 ± 10.51	59.25 ± 10.20	*t* = 0.486; *p* = 0.628
Age of onset (years)^a^	55.75 ± 10.57	–	–
Hoehn and Yahr stage^a^	2.37 ± 0.77	–	–
UPDRS III score^a^	31.30 ± 12.43	–	–
Disease duration (years)^a^	4.44 ± 3.61	–	–

*^a^Values are expressed as the mean ± standard deviation. PD, Parkinson’s disease; UPDRS, Unified Parkinson’s Disease Rating Scale.*

### CircRNAs Are Differentially Expressed in the Peripheral Blood of Patients With Parkinson’s Disease and Have the Potential as Biomarkers for Parkinson’s Disease

A total of 10 candidate circRNAs (five upregulated and five downregulated) were retrieved for further verification in an independent cohort of 63 patients with PD and 60 healthy controls. The clinical characteristics of this cohort are described in Table 1. The mean age was 60.16 ± 10.51 years for the PD group and 59.25 ± 10.20 years for controls. Statistical analyses showed no significant difference in sex or age between both groups (χ^2^ = 0.004, *p* = 0.951; *t* = 0.486, *p* = 0.628, respectively). The average UPDRS III score was 31.30 ± 12.43 for the PD group, and the disease duration was 4.44 ± 3.61 years. Enrolled patients had an average age of onset of 60.16 ± 10.51 years, and the mean clinical Hoehn and Yahr stage was 2.37 ± 0.77.

**FIGURE 2:**
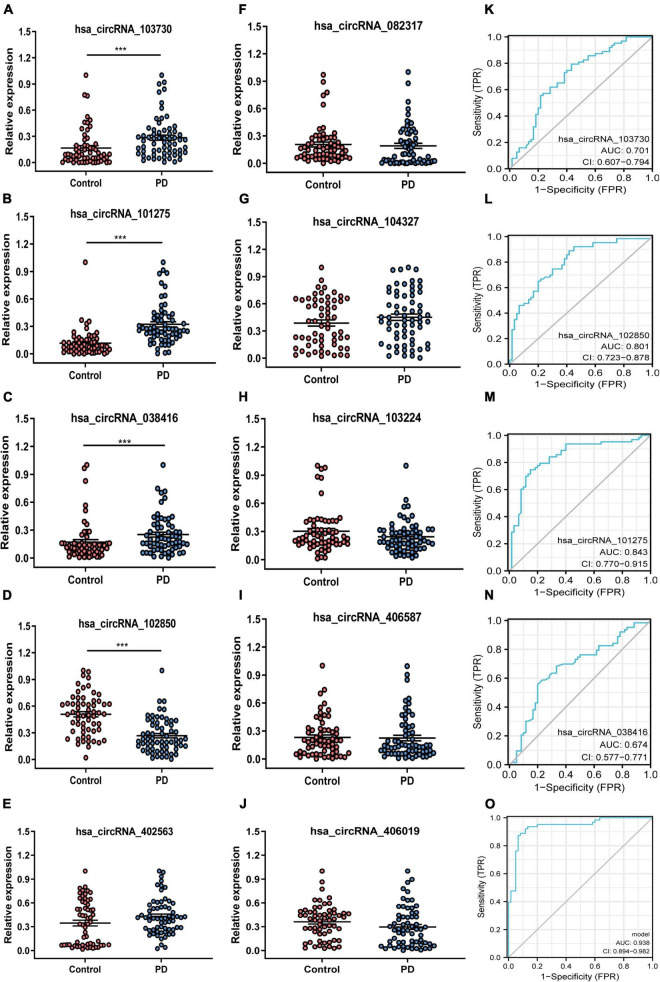
Expression profile and ROC curves for circRNAs in patients with PD and healthy controls peripheral blood sample. **(A–J)** The relative expression level of ten candidate circRNAs in patients with PD and healthy control using RT-qPCR detection. **(K–O)** ROC analysis of the four differentially expressed circRNAs in peripheral blood samples. PD, Parkinson’s disease; control, healthy controls; circRNA, circular RNA; hsa, homo sapiens; AUC, the area under the curve; TPR, true positive rate; FPR, false-positive rate. ***p-value < 0.001.

The ten candidate circRNAs were verified using divergent primers rather than the standard convergent primers *via* RT-qPCR. Of the 10 circRNAs, 4 were confirmed as differentially expressed between both groups (Figures 2A–J). The circRNAs hsa_circRNA_103730, hsa_circRNA_101275, and hsa_circRNA_038416 (Figures 2A–C) were significantly upregulated, while hsa_circRNA_102850 (Figure 2D) was downregulated in patients with PD when compared to healthy controls. Considering that the incidence of male patients with PD is greater than female patients, we performed a stratification analysis based on sex. However, the results did not show a significant difference between male and female patients with PD (Supplementary Figure 1A–J).

The diagnostic value of the differentially expressed circRNAs in PD was performed using the ROC curve. The AUC for hsa_circRNA_103730, hsa_circRNA_102850, hsa_circRNA_101275, and hsa_circRNA_038416 for PD was 0.701, 0.801, 0.843, and 0.674, respectively (2K–N). A circRNA panel combining the four candidate circRNAs showed a higher diagnostic ability to distinguish patients with PD from healthy controls (AUC = 0.938) (Figure 2O).

### CircRNA–miRNA–mRNA Interaction Network Construction and Transcription Factor–circRNA Interaction Prediction

**FIGURE 3:**
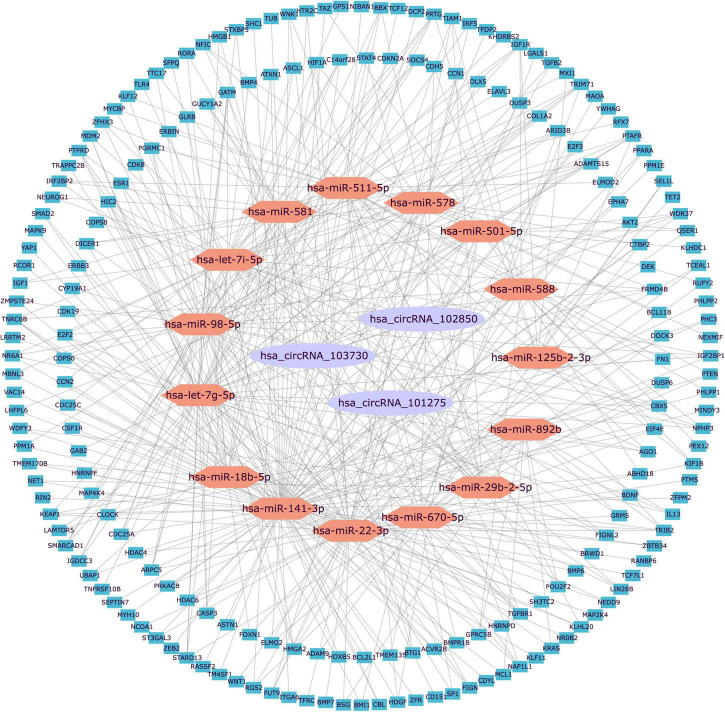
The visualization of the circRNA–miRNA–mRNA regulation network. The circRNA–miRNA–mRNA network was constructed based on three circRNAs, 15 miRNAs, and 200 mRNAs. circRNAs were plotted as purple ellipses, miRNAs were plotted as orange hexagons, and mRNAs were plotted as blue squares. miR, microRNA; circRNA, circular RNA.

Generally, ncRNAs act as competing endogenous RNAs (ceRNAs) that regulate target RNA transcripts by binding competitively to shared miRNAs. It has been well established that circRNAs have many miRNA-binding sites that may act as a ceRNA sponge for miRNAs. Considering the circRNA with an AUC > 0.70 was considered with sufficient specificity and sensitivity, therefore, hsa_circRNA_102850, hsa_circRNA_101275, and hsa_circRNA_103730 were selected for the circRNA-regulatory network prediction. Interactions between the three candidate circRNAs and their relationships with miRNAs and target genes were predicted by building a unified interaction-network model. The interactions between circRNAs and miRNAs were predicted by the miRNA target prediction software. The top five putative miRNAs of the three candidate circRNAs were selected to predict target genes, and the top 200 target genes were used to construct the circRNA–miRNA–mRNA interaction networks (Figure 3). The results indicated that the candidate circRNAs may act as endogenous RNAs for modulating target gene expression.

The TRCirc was used to establish a transcription factor–circRNA (TF–circRNA) network to explore the precise molecular mechanism of transcriptional regulation of the candidate circRNAs. Hsa_circRNA_103730 (circBase ID: hsa_circ_0005654) and hsa_circRNA_101275 (circBase ID: hsa_circ_0030428) were predicted to be regulated by targeting TH-relevant transcription factors such as *GATA2* and *GATA3* (Polanski et al., 2010).

### Gene Ontology and Kyoto Encyclopedia of Genes and Genomes Enrichment Analysis of the Differentially Expressed circRNAs

**FIGURE 4:**
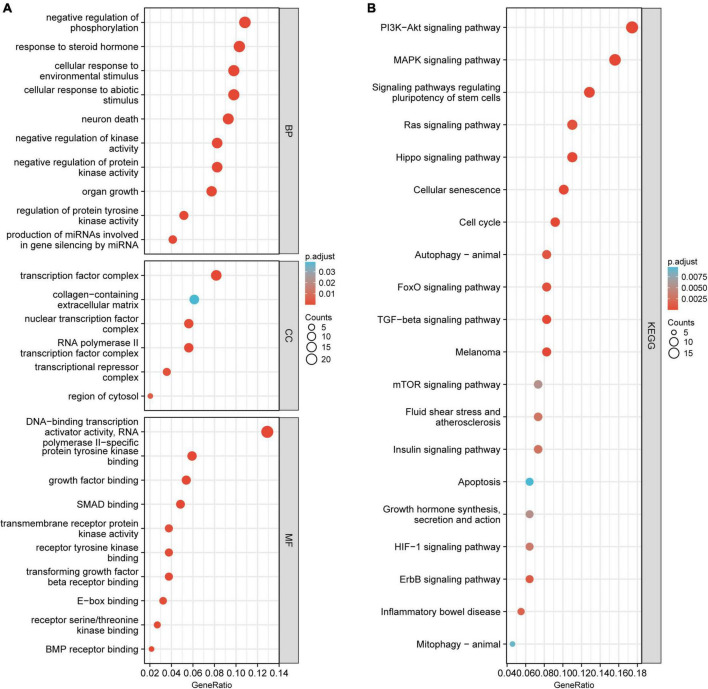
The GO and KEGG pathway enrichment analysis for 200 mRNAs. **(A)** The major enriched and meaningful GO terms. **(B)** The top 20 enrichment scores in the KEGG pathway analysis. GO, Gene Ontology; BP, biological processes; CC, cellular components; MF, molecular functions; KEGG, Kyoto Encyclopedia of Genes and Genomes.

The GO and KEGG enrichment analyses were conducted to investigate the biological function of the candidate circRNAs. The GO analysis indicated that the 200 mRNAs were mainly enriched in negative regulation of phosphorylation, cellular response to environmental stimulus, response to a steroid hormone, transcription factor complex, collagen-containing extracellular matrix, and nuclear transcription factor complex (Figure 4A). The PI3K–Akt signaling pathway and MAPK signaling pathway were found to have significant enrichment in the KEGG pathway analysis (Figure 4B).

### Protein–Protein Interaction Network Construction

**FIGURE 5:**
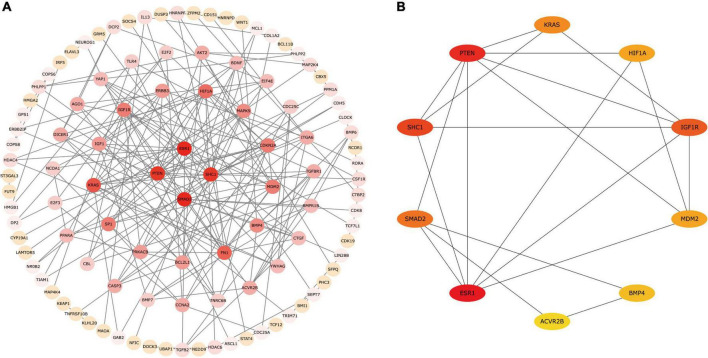
The PPI network for 200 mRNAs. **(A)** A PPI network composed of 107 proteins and 200 edges. **(B)** The sub-network containing 10 hub genes. Proteins are plotted as circles and given different colors, the darker the color, the more important the protein is in the interaction network. PPI, protein–protein interaction.

We constructed a PPI network according to circRNA–miRNA–mRNA interaction analysis. The PPI networks identified 107 proteins and 200 edges of the candidate circRNA networks that may be involved in PD (Figure 5A). We used the cytoHubba plugin to find the critical genes in the process of PD pathogenesis and obtained a sub-network containing 10 genes, including *ESR1, PTEN, SHC1, IGF1R, SMAD2, KRAS, MDM2, HIF1A, BMP4*, and *ACVR2B* (Figure 5B). The results of PPI interaction networks showed that *ESR1, PTEN*, and *SHC1* may be involved in PD as the most important targets.

## Discussion

Parkinson’s disease (PD) is an irreversible neurodegenerative disease (Hayes, 2019). The precise pathogenic mechanisms underlying PD remain largely unknown. Many studies have been undertaken in recent years to provide new insights into PD but mainly focus on protein-coding genes or miRNAs (Goh et al., 2019; Rathore et al., 2021). CircRNAs have attracted much attention for their special characteristics such as conserved, tissue- or cell-type specific, and involvement with miRNA regulation in several disorders such as cardiovascular diseases (Altesha et al., 2019), cancer (Chen and Shan, 2021), and diabetes (Sakshi et al., 2021), but their roles in PD remain unclear.

In this study, we first used microarray analyses to obtain the expression profiles of circRNAs in patients with PD and healthy controls. A total of 139 dysregulated circRNAs were identified. Differential expression analysis in an independent PD cohort revealed that hsa_circ_101275, hsa_circ_038416, hsa_circ_103730, and hsa_circ_12850 might be used as potential novel biomarkers for PD. Besides, a circRNA panel combining the above four differentially expressed circRNA showed a higher diagnostic ability to distinguish patients with PD from controls (AUC = 0.938). Although a previous study has identified that some circRNAs were differentially expressed in patients with PD and controls (Zhong et al., 2021), the networks and underlying mechanisms involved in circRNA-mediated regulation of PD have not been explored.

To investigate the potential function of the differentially expressed circRNAs in PD, we constructed a core circRNA–miRNA–mRNA regulatory network, protein–protein interaction, and transcription factor–circRNA interaction analyses. The GO and KEGG analyses showed that these candidate circRNAs were enriched in PI3K–Akt signaling and MAPK signaling pathways. The disruption of Akt-mediated signal transduction contributes to the pathogenesis of several neurodegenerative diseases (Rai et al., 2019), including PD (Cao et al., 2017; Chen et al., 2017; Hu et al., 2018). For instance, a recent study has indicated that amentoflavone protects dopaminergic neurons against MPTP/MPP+-induced neurotoxicity, possibly by activating the PI3K/Akt signaling pathways (Cao et al., 2017). Whether these candidate circRNAs are involved in PD through the PI3K–Akt signaling pathway needs to be further investigated.

Generally, circRNAs serve as miRNA sponges, adsorbing miRNAs and altering miRNA expression levels, hence influencing downstream target gene expression (Moreno-García et al., 2020). Therefore, we constructed circRNA–miRNA–mRNA regulatory networks and identified several miRNAs such as miR-29b-2-5p, miR-22-3P, miR-141-3p, and miR-18b-5p in the candidate circRNA–miRNA–mRNA regulatory networks. What needs to be noted is that one of the most important PD pathogenic gene *GBA* (Riboldi and Di Fonzo, 2019) and its pseudogene *GBAP1* are miR-22-3P target genes (Straniero et al., 2017). Another study found miR-22 was an important target for suppressing apoptosis and the generation of reactive oxygen species in a cellular model of PD (Yang et al., 2016). Whether these candidate circRNAs can act as a sponge for miR-29b-2-5p and miR-22-3P by modulating the GBA axis and the apoptosis cascade should be further explored.

We established circRNA–miRNA–mRNA regulatory networks and identified 10 hub genes (*ESR1, PTEN, SHC1, IGF1R, SMAD2, KRAS, MDM2, HIF1A, BMP4*, and *ACVR2B*) that were closely related to PD by using PPI network analysis. The identified hub genes might provide important targets in the pathogenesis of PD. For example, genetic studies have found *ESR1* gene polymorphisms may be linked to PD susceptibility (Handel et al., 2013), *IGF1R* was discovered to have anti-inflammatory properties in a rat model of PD (Xu et al., 2009; Du et al., 2021), *BMP4* can induce the phenotypic of striatal dopaminergic neurons *in vitro* (Stull et al., 2001). Overall, the findings further support the importance of the identified circRNAs in PD, though the exact mechanisms are still unclear.

At present, circRNA studies are mainly focused on its downstream mechanisms, but upstream mechanisms, such as how circRNAs are generated, are still lacking. Transcription factor *Twist1* has been identified to transcriptionally regulate the expression of circRNA-10720 (Meng et al., 2018), and another transcription factor *c-Myc* could accelerate the circularization and biogenesis of circUHRF1 (Zhao et al., 2020), suggesting that transcription factors may be involved in the circRNA upstream regulation process. We used TRCirc to establish a transcription factors–circRNA (TF-circRNA) network and found hsa_circRNA_103730 and hsa_circRNA_101275 were predicted to be regulated by TH-relevant transcription factors such as *GATA2* and *GATA3* (Polanski et al., 2010), which further expanded the function of the two PD-related transcription factors.

In summary, our results suggest the circRNA panel (hsa_circ_101275, hsa_circ_038416, hsa_circ_103730, and hsa_circ_12850) and the established circRNA–miRNA–mRNA regulation networks might provide potential novel biomarkers and therapeutic targets for PD.

## Data Availability Statement

The datasets presented in this study can be found in online repositories. The names of the repository/repositories and accession number(s) can be found in the article/Supplementary Material.

## Ethics Statement

The studies involving human participants were reviewed and approved by the Ethics Committee of The First Affiliated Hospital of Guangxi Medical University. The patients/participants provided their written informed consent to participate in this study.

## Author Contributions

YX and JJL wrote the article and worked with HC on the conception and design. QZ and HH performed the experiments. JL and JH analyzed the data. QO and JW polished the article. All authors contributed to the article and approved the submitted version.

## Conflict of Interest

The authors declare that the research was conducted in the absence of any commercial or financial relationships that could be construed as a potential conflict of interest.

## Publisher’s Note

All claims expressed in this article are solely those of the authors and do not necessarily represent those of their affiliated organizations, or those of the publisher, the editors and the reviewers. Any product that may be evaluated in this article, or claim that may be made by its manufacturer, is not guaranteed or endorsed by the publisher.

## Footnotes

1
http://www.licpathway.net/TRCirc/view/index

## Supplementary Material

Supplementary Figure 1 Relative expression of candidate circRNAs in male patients with PD and female patients with PD. PD, Parkinson’s disease; control, healthy controls; circRNA, circular RNA; hsa, homo sapiens. The stratification analyses of the ten candidate circRNAs in patients with PD and healthy control according to sex (Supplementary Figure 1A–J).
