# Supplementary figures and images for: The Potential Circular RNAs Biomarker Panel and Regulatory Networks of Parkinson’s Disease
Source: Front Neurosci. 2022 May 13;16:893713. doi: 10.3389/fnins.2022.893713 (PMC9136065; doi:10.3389/fnins.2022.893713)

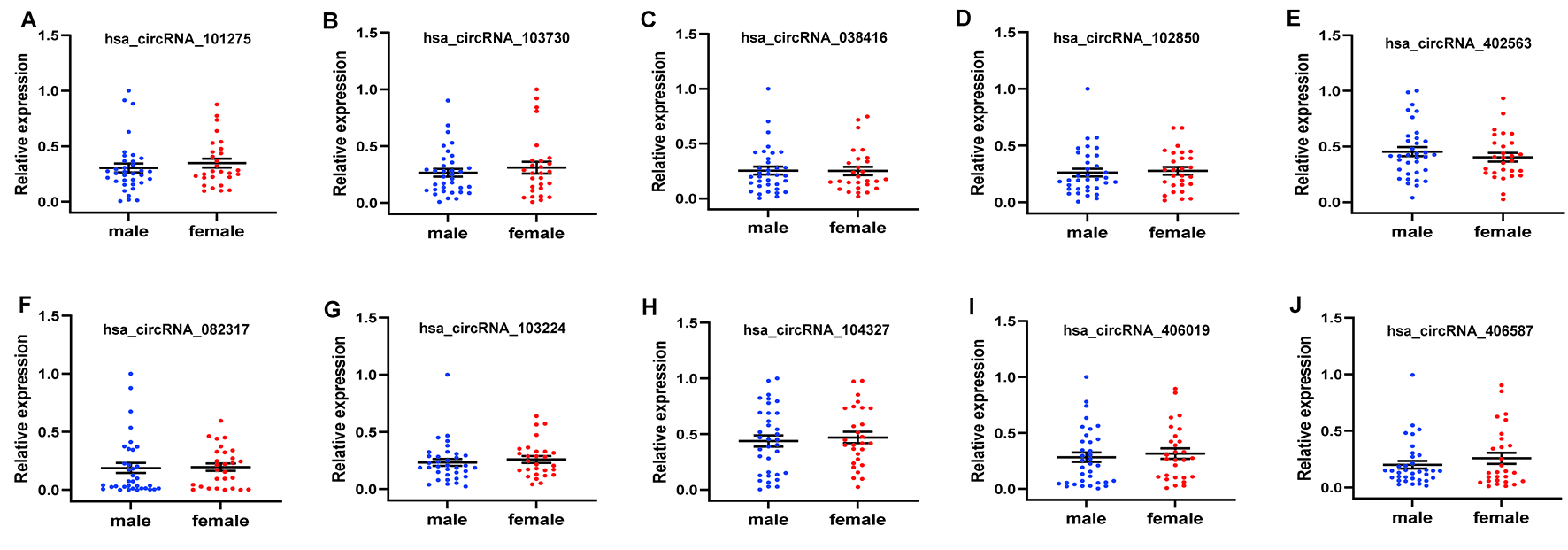

Supplement: Supplementary Figure 1 — Relative expression of candidate circRNAs in male patients with PD and female patients with PD. PD, Parkinson’s disease; control, healthy controls; circRNA, circular RNA; hsa, homo sapiens. The stratification analyses of the ten candidate circRNAs in patients with PD and healthy control according to sex (Supplementary Figure 1A–J). [file Image_1.TIF]
