# Supplementary material for: The Potential Circular RNAs Biomarker Panel and Regulatory Networks of Parkinson’s Disease
Source: Front Neurosci. 2022 May 13;16:893713. doi: 10.3389/fnins.2022.893713 (PMC9136065; doi:10.3389/fnins.2022.893713)
Supplement: Supplementary file 2 [file Table_1.DOCX]

Table S1 Real-time quantitative PCR primer sequences.

| Genes | Primer sequence (5'→3') |
| --- | --- |
| hsa_circRNA_038416 | F: TATTCCTGTTTTGGAGACTGCCTAT |
|  | R: TACAGCAACAACCCCTATTAGTGAG |
| hsa_circRNA_101275 | F: GGCAGCTGTGTGAAGCCAAG |
|  | R: TCACCAGCTTGGCTCTTGGT |
| hsa_circRNA_406019 | F: ACCTTGTGATCTGCCCACCT |
|  | R: TCCTGAATGGATTTCTTCACTGGTT |
| hsa_circRNA_104327 | F: TGCCCAGTGTTTCTCTTCAGTCA |
|  | R: ACTCACTTTGTGGCTCCTGGA |
| hsa_circRNA_082317 | F: CACCATATGAAGGCGGAGTATGGA |
|  | R: TCCTCCCAGGATCGTAACCTCA |
| hsa_circRNA_402563 | F: TCGTCAGCCCAAACATCTCCT |
|  | R: AGGTCATCCTCTGGTTCAGTTGG |
| hsa_circRNA_102850 | F: ACTGGAACTGTAGGAGAGGCTC |
|  | R: TCCCGTAGTTGCTGATATACGAAG |
| hsa_circRNA_103224 | F: GGCGTATCTCCTGCCTGCAA |
|  | R: CCAACGGAAATCCCTGGCAC |
| hsa_circRNA_103730 | F: TGAGCGTCCCTATCAATGTCCT |
|  | R: AGGCTTGATGCTGAAGAACACT |
| hsa_circRNA_406587 | F: AGTCTGCCTGCTTTTGACCCT |
|  | R: TGCCACTGCCGACTGGTATC |
| GAPDH | F: GGGAGCCAAAAGGGTCAT |
|  | R: GAGTCCTTCCACGATACCAA |

Has, Homo sapiens; circRNA, circular RNA; F, forward; R, reverse; GAPDH, Glyceraldehyde 3-phosphate dehydrogenase.
